# Massively Reconstructing Hydrogen Bonding Network and Coordination Structure Enabled by a Natural Multifunctional Co‐Solvent for Practical Aqueous Zn‐Ion Batteries

**DOI:** 10.1002/advs.202400336

**Published:** 2024-04-11

**Authors:** Yuanze Yu, Qian Zhang, Pengfei Zhang, Xu Jia, Hongjiang Song, Shengkui Zhong, Jie Liu

**Affiliations:** ^1^ Youth Innovation Team of Shandong Higher Education Institutions College of Chemical Engineering Qingdao University of Science and Technology Qingdao Shandong 266042 P. R. China; ^2^ Weifang Key Laboratory of Green Processing of Separator for Chemical Power Sources School of Chemistry and Engineering Weifang Vocational College Weifang Shandong 261108 P. R. China; ^3^ College of Marine Science and Technology Yazhou Bay Innovation Research Institute Hainan Tropical Ocean University Sanya Hainan 572022 P. R. China

**Keywords:** aqueous Zn‐ion batteries, coordination structure, electrolyte design, hydrogen bonding network, practical operation condition

## Abstract

The practical application of aqueous Zn‐ion batteries (AZIBs) is hindered by the crazy Zn dendrites growth and the H_2_O‐induced side reactions, which rapidly consume the Zn anode and H_2_O molecules, especially under the lean electrolyte and Zn anode. Herein, a natural disaccharide, d‐trehalose (DT), is exploited as a novel multifunctional co‐solvent to address the above issues. Molecular dynamics simulations and spectral characterizations demonstrate that DT with abundant polar −OH groups can form strong interactions with Zn^2+^ ions and H_2_O molecules, and thus massively reconstruct the coordination structure of Zn^2+^ ions and the hydrogen bonding network of the electrolyte. Especially, the strong H‐bonds between DT and H_2_O molecules can not only effectively suppress the H_2_O activity but also prevent the rearrangement of H_2_O molecules at low temperature. Consequently, the AZIBs using DT30 electrolyte can show high cycling stability even under lean electrolyte (E/C ratio = 2.95 µL mAh^−1^), low N/P ratio (3.4), and low temperature (−12 °C). As a proof‐of‐concept, a Zn||LiFePO_4_ pack with LiFePO_4_ loading as high as 506.49 mg can be achieved. Therefore, DT as an eco‐friendly multifunctional co‐solvent provides a sustainable and effective strategy for the practical application of AZIBs.

## Graphical abstract

An effective and economical electrolyte design is proposed by introducing DT as the co‐solvent of the electrolyte and the hydrogen bonding network of the electrolyte is massively reconstructed. The reversibility and stability of the Zn anode are significantly improved, and high cycling stability is achieved even under lean electrolyte (E/C ratio = 2.95 µL mAh^−1^), low N/P ratio (3.4), and low temperature (−12 °C).

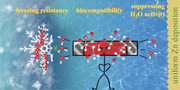

## 1 Introduction

Rapid developments in electronics, transportation, and industry have accelerated the demand for high‐performance energy storage devices. In addition to conventional Li‐ion batteries,^[^ ^1^, ^2^, ^3^ ^]^ novel alkali metal ion batteries (K, Na) and multivalent metal ion batteries (Zn, Mg, and Al) have also been extensively studied.^[^ ^4^, ^5^, ^6^, ^7^, ^8^, ^9^, ^10^, ^11^, ^12^ ^]^ Among them, aqueous Zn‐ion batteries (AZIBs) are considered as one of the most promising energy storage systems, owing to their high safety, environmental friendliness, and abundant zinc resources.^[^ ^13^, ^14^, ^15^ ^]^ In addition, the Zn anode has a low redox potential (0.762 V vs. SHE) and a high theoretical volumetric capacity (5855 mAh cm^−3^).^[^ ^16^, ^17^ ^]^ Despite these advantages, the development of AZIBs is still severely hindered by the uneven Zn deposition and the active H_2_O in the electrolyte. The uneven Zn deposition will lead to the frantic growth of Zn dendrites, which will puncture the separator resulting in short‐circuiting and failure of cell. The extensive active H_2_O molecules undergo a hydrogen evolution reaction (HER) during the Zn deposition process, leading to cell bloating and security issues. At the same time, the H_2_O molecules spontaneously react with Zn electrode, leading to electrode corrosion and active material loss.^[^ ^18^, ^19^ ^]^ In addition, the freezing of aqueous electrolyte below zero temperature also limits the practical application of the AZIBs.

Many efforts have been made to improve the electrochemical performance of AZIBs, including the optimization of the Zn anode surface,^[^ ^20^, ^21^, ^22^, ^23^ ^]^ the design of alloying anode,^[^ ^24^, ^25^, ^26^ ^]^ the separator functionalization,^[^ ^27^, ^28^ ^]^ and the optimization of electrolytes.^[^ ^29^, ^30^, ^31^, ^32^ ^]^ Among them, the electrolyte optimization is an economical and effective method and has been widely studied. Exploiting functional additives,^[^ ^33^, ^34^ ^]^ adjusting Zn salt type,^[^ ^35^, ^36^ ^]^ introducing co‐solvent,^[^ ^37^, ^38^ ^]^ constructing eutectic solvents,^[^ ^39^, ^40^ ^]^ and concentrated electrolytes,^[^ ^41^, ^42^ ^]^ have been demonstrated as promising approaches. For example, tripropylene glycol was used as an electrolyte additive to modulate the coordination structure of Zn^2+^ ions and to construct an adsorption layer on the Zn anode, which improved the reversibility of the Zn anode.^[^ ^34^ ^]^ Zn(CH_3_SO_3_)_2_ electrolyte was introduced into AZIBs to decrease H_2_O molecules in the coordination structure of Zn^2+^ ions, inhibiting the formation of Zn dendrites and realizing a long cycle time of over 800 h in Zn||Zn cell.^[^ ^43^ ^]^ Ethylene glycol was introduced into the aqueous electrolyte as a co‐solvent to destroy the crystal structure of H_2_O under low temperature, thus the Zn||PANI‐V_2_O_5_ cell was cycled stably at −20 °C for more than 110 days.^[^ ^29^ ^]^ The concentrated hybridized (4 M Zn(CF_3_SO_3_)_2_ + 2 m LiClO_4_) electrolyte formed a passivation layer on the surface of the Zn anode, which is conducive to uniform current distribution, and realizes dendrite‐free Zn plating/stripping, with a capacity retention rate of more than 90% after 285 cycles in Zn||LiFePO_4_ full cell.^[^ ^41^ ^]^ Most of the electrolyte design has effectively enhanced the uniformity and reversibility of Zn deposition. However, the H_2_O‐induced issues still remain many challenges for the practical application of AZIBs, especially under lean electrolyte, low N/P ratio, and low temperature.

d‐trehalose (DT) is a natural disaccharide, which can significantly enhance the frost and drought resistance of natural creature.^[^ ^44^ ^]^ Recently, DT has been demonstrated to inhibit the Zn dendrites growth as an electrolyte additive (5 mm).^[^ ^45^ ^]^ However, the common disadvantage of most additives is that they are inevitably consumed during cells cycling,^[^ ^46^ ^]^ especially at lean electrolyte and large capacity. Moreover, the tiny additives can only reconstruct localized hydrogen bonding (H‐bonding) network and coordination structure. Herein, because it is environmentally friendly and economical, DT, for the first time, is exploited as a massive co‐solvent (0.88 m) to improve the reversibility and utilization of Zn anodes and decrease the H_2_O‐induced side reactions under practical working conditions. Molecular dynamics (MD) simulations demonstrate that DT can modulate the coordination structure of Zn^2+^ ions, and thus regulates the deposition behavior of Zn. Meanwhile, DT with abundant polar −OH groups shows strong affinity with H_2_O molecules and massively reconstructs the H‐bonding network of electrolyte. The robust H‐bonds between DT and H_2_O can not only effectively suppress the H_2_O activity and reduce the H_2_O depletion, but also destroy the crystal structure of H_2_O under low temperature to hinder the freezing of the electrolyte. As a result, the AZIBs can successfully operate under harsh conditions (lean electrolyte of 2.95 µL mAh^−1^, low N/P ratio of 3.4, and low temperature of −12 °C). As a proof‐of‐concept, a Zn||LiFePO_4_ pack with LiFePO_4_ loading of 506.49 mg can be achieved. Therefore, the biocompatible DT as a massive co‐solvent can be applied to achieve practical AZIBs and reduce originally the pollution of waste batteries, simultaneously.

## 2 Results and Discussion

**Figure 1:**
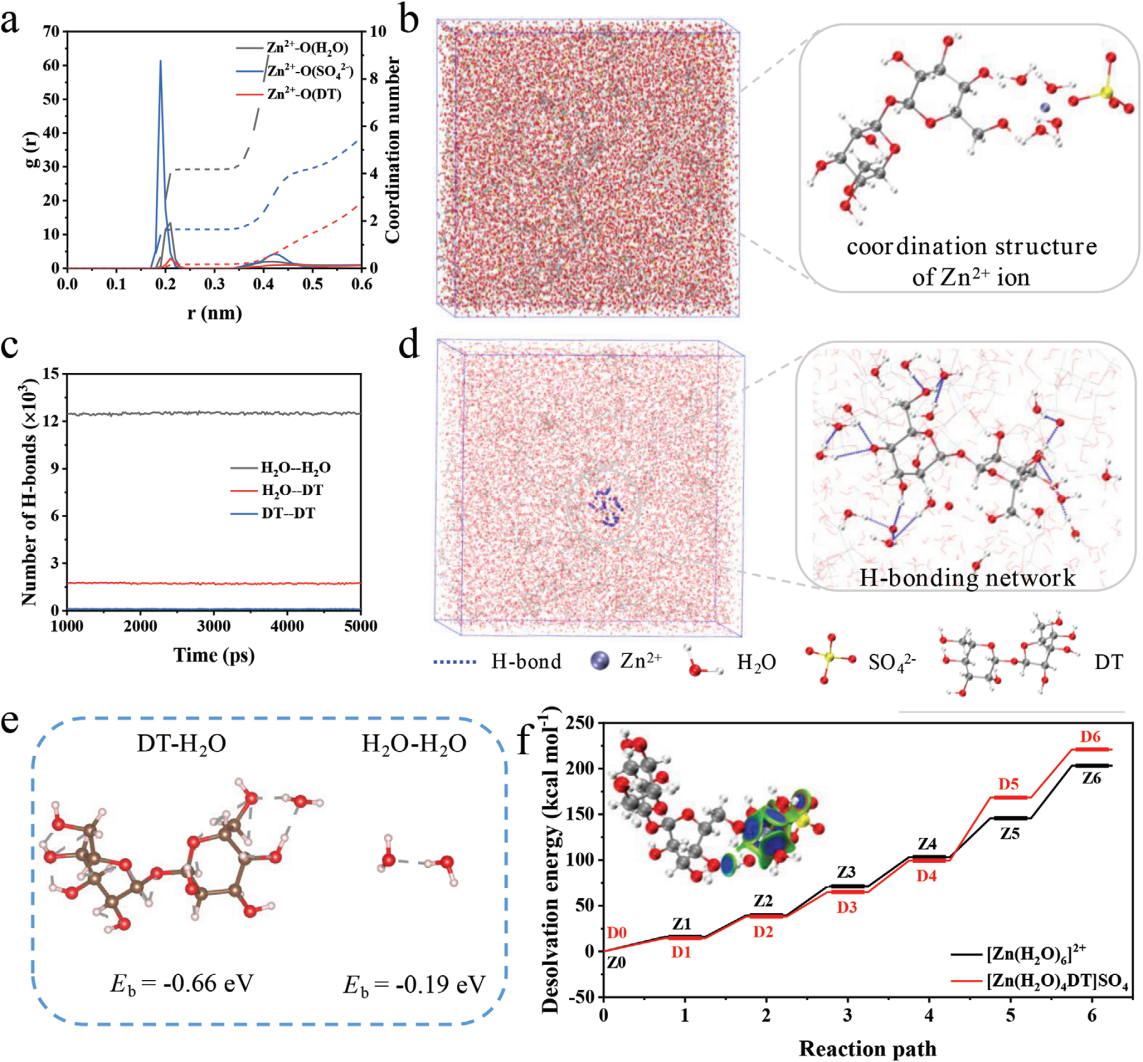
Theoretical simulations of DT30: a) The RDF and CN plots for DT30 electrolyte. b) Snapshots of the Zn^2+^ ion coordination structure in DT30. c) The number of different H‐bonds in DT30. d) Snapshots of the H‐bonding network in DT30. e) Binding energy of H_2_O with DT and H_2_O. f) Calculated desolvation energy barriers for [Zn(H_2_O)_4_DT]SO_4_ and [Zn(H_2_O)_6_]^2+^ (the inset shows the interaction visualization model of [Zn(H_2_O)_4_DT]SO_4_, with strong interactions in blue and weak interactions in green).

DT is a non‐reducing natural disaccharide composed of two glucose molecules (Figure S1, Supporting Information), widely used in medicine, food, agriculture, and other aspects. The abundant polar −OH groups in DT make it a facile platform to form strong interactions with polar groups/molecules. MD simulations were performed for DT30 to theoretically investigate the interactions of DT with Zn^2+^ ions as well as H_2_O molecules. **Figure** **1**a illustrates the radial distribution function (RDF) and coordination number (CN) of Zn^2+^‐O. There are three coordination structures of the Zn^2+^ ion, namely Zn^2+^‐O (H_2_O), Zn^2+^‐O (SO_4_^2−^), and Zn^2+^‐O (DT). As shown in Table S1 (Supporting Information), the CN of Zn^2+^‐O (DT) is simulated to be 0.17, which suggests that DT is successfully involved in the coordination structure of Zn^2+^ ions. Consequently, the DT molecule replaces and decreases the H_2_O molecules distributed around the Zn^2+^ ion, and a new coordination structure of the Zn^2+^ ion emerges in DT30 (Figure 1b; Figure S2, Supporting Information). As shown in Figure 1f, the desolvation energy barrier of [Zn(H_2_O)_4_DT]SO_4_ solvation sheath is increased to 220.95 kcal mol^−1^ due to the strong interaction between DT and Zn^2+^ ions, which benefits the uniform deposition. This plays an important role in regulating Zn deposition behavior and decreasing the Zn nucleation size. Three forms of H‐bonds in the DT‐containing electrolyte can be present, including those between H_2_O molecules, between H_2_O and DT molecules, and between DT molecules. As shown in Figure 1c and Table S2 (Supporting Information), the simulation results display a large amount of H‐bonds between DT and H_2_O molecules, numbering ≈3 × 10^3^. However, there are almost no H‐bonds between DT molecules, suggesting the DT molecules are surrounded by H_2_O molecules (Figure 1d). Density functional theory (DFT) calculations were performed to investigate the interaction behavior between DT and H_2_O molecules. The calculated binding energy of DT‐H_2_O (‐0.66 eV) is significantly higher than that of H_2_O‐H_2_O (‐0.19 eV) (Figure 1e). These results powerfully demonstrate that DT as a co‐solvent can form strong interactions with H_2_O molecules to break the intrinsic H‐bonds between H_2_O molecules and reconstruct the H‐bonding network in the electrolyte. This effectively hinders the orderly arrangement of H_2_O molecules, lowering the freezing point of the aqueous electrolyte.^[^ ^47^ ^]^ At the same time, the strong H‐bonds between DT and H_2_O molecules can effectively anchor the H_2_O molecules to reduce the active H_2_O on the Zn/electrolyte interfaces, and thus suppress the corrosion of the Zn anode and HER.^[^ ^48^, ^49^ ^]^

**Figure 2:**
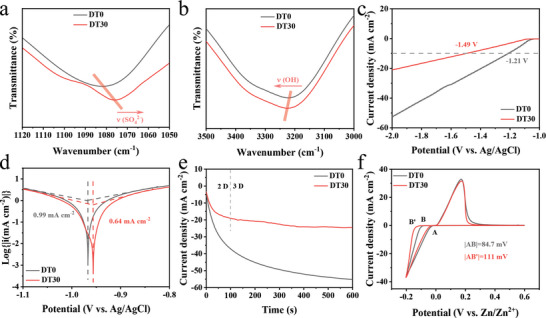
a,b) FTIR spectra of ZnSO_4_ electrolytes with/without DT. c) LSV curves and d) Tafel plots in DT0 and DT30 using a three‐electrode system. e) CA curves of Zn||Zn symmetric cells in DT0 and DT30. f) CV curves of Zn||Ti asymmetric cells in DT0 and DT30.

Fourier transform infrared (FTIR) spectra were applied to further illustrate the effect of DT on the coordination structure of Zn^2+^ ions and the H‐bonding network of H_2_O (full FTIR spectra displayed in Figure S3, Supporting Information). The absorption peak of ν(SO_4_^2−^) in **Figure** **2**a is shifted from 1082.4 to 1076.1 cm^−1^ after introducing the DT co‐solvent, which represents a decrease in the number of free SO_4_^2−^ ions in the electrolyte. It suggests that the H‐bonds between DT and H_2_O molecules weaken the interaction between H_2_O molecules and Zn^2+^ ions, allowing the SO_4_^2−^ ion to enter the primary solvation sheath.^[^ ^50^ ^]^ This is consistent with the results of MD. After the addition of DT, the stretching vibration of −OH bonds in H_2_O molecules shifts from 3218.1 cm^−1^ to a higher wavenumber of 3225.8 cm^−1^ (Figure 2b), indicating a decrease in H‐bonds between H_2_O molecules.^[^ ^51^ ^]^ The local environment of ^1^H from H_2_O in different electrolytes was analyzed by ^1^H nuclear magnetic resonance (NMR) spectroscopy (Figure S4, Supporting Information). In ZnSO_4_ aqueous solution, the ^1^H chemical shift peak moves to a higher electric field of 4.72, due to the fact that the [Zn(H_2_O)_6_]^2+^ clusters occur and weaken the electron density around the protons in the H_2_O molecule.^[^ ^52^ ^]^ The ^1^H chemical shift in electrolyte containing DT moves to the lower electric field position of 4.68, suggesting the broken and decreasing H‐bonds between H_2_O molecules, in agreement with the FTIR results.^[^ ^38^ ^]^ The differential scanning calorimeter (DSC) tests were used to investigate the anti‐freezing performance of the electrolyte using DT as a co‐solvent, as displayed in Figure S5 (Supporting Information). The freezing point of the electrolyte containing DT30 is −19.85 °C, which is much lower than that of DT0 (‐11.54 °C). As the temperature drops below freezing point, the disordered H_2_O molecules rearrange themselves into a solid state, leading to freezing.^[^ ^53^ ^]^ However, after adding DT, the strong H‐bonds form between DT and H_2_O molecules can prevent the reordering of H_2_O molecules, thus lower the freezing point of the electrolyte. Figure S6 (Supporting Information) visually displays the photograph of the DT0 and DT30 under −12 °C, in which the DT0 has been frozen while the DT30 remains fluidity.

In DT30, the suppressed side reactions such as HER and corrosion of the Zn anode were also experimentally demonstrated. Linear sweep voltammetry (LSV) curves (Figure 2c) show that the HER potential in DT30 increased by 280 mV compared to DT0 at a current density of 10 mA cm^−2^. Furthermore, the presence of DT raises the corrosion voltage from −0.967 to −0.956 V and obviously lowers the corrosion current density from 0.99 to 0.64 mA cm^−2^ (Figure 2d). Scanning electron microscopy (SEM) images of the Zn electrode were obtained to directly observe the uniform deposition of Zn and suppressed corrosion of the electrode in DT30. The surface of the Zn electrode after 50 cycles in the Zn||Zn symmetric cell containing DT0 becomes rough and multitudinous hexagonal by‐products are produced (Figure S7a, Supporting Information). On the contrary, the surface of the Zn electrode in DT30 is very flat without by‐products (Figure S7b, Supporting Information). X‐ray diffraction (XRD) pattern is shown in Figure S8 (Supporting Information), the diffraction peaks of Zn_4_SO_4_(OH)_6_·5H_2_O by‐products in DT30 electrolyte are obviously weakened, which indicates that the HER is inhibited by DT. In addition, the addition of DT facilitates the deposition of Zn on the beneficial (002) surface, which may help to achieve dendrite‐free Zn deposition.^[^ ^54^ ^]^ These results powerfully indicate that the strong interactions between DT and H_2_O molecules occur, and thus effectively decrease active H_2_O molecules and avoid H_2_O‐induced side reactions.

The chronoamperometry (CA) curves were used to investigate the Zn deposition behavior in different electrolytes. As shown in Figure 2e, the current density of the Zn||Zn symmetric cell in DT0 continues to decrease with time, which implies a 2D expansion of the effective electrode area, corresponding to the formation of Zn dendrites.^[^ ^55^ ^]^ On the contrary, the current density of the Zn||Zn symmetric cell in DT30 is much more stable after 100 s, suggesting that DT promotes the uniform deposition of Zn. Cyclic voltammetry (CV) curves were obtained to further study the electrochemical deposition process of Zn. Figure 2f shows that the nucleation over‐potential of Zn in DT30 is appropriately increased by 26.3 mV compared to that of DT0. The same phenomenon is also observed in the time‐voltage curves of Zn||Cu asymmetric cells (Figure S9, Supporting Information). This is due to the increased desolvation energy barrier. The larger nucleation over‐potential can lead to a smaller nucleation radius, resulting in a fine and smooth Zn deposition.^[^ ^56^, ^57^, ^58^ ^]^ In‐situ optical microscopy visualizes more uniform Zn deposition in DT30 (Figure S10, Supporting Information).

**Figure 3:**
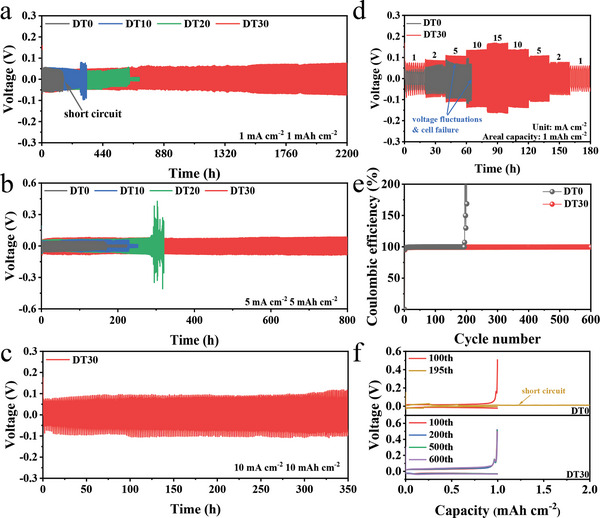
Electrochemical performance evaluation of half cells at room temperature: GCD curves of Zn||Zn symmetric cells in different electrolytes at a) 1 mA cm^−2^/1 mAh cm^−2^ and b) 5 mA cm^−2^/5 mAh cm^−2^. c) Cycling performance of Zn||Zn symmetric cell in DT30 at 10 mA cm^−2^/10 mAh cm^−2^. d) Rate performance of Zn||Zn symmetric cells in DT0 and DT30. e) CE and f) corresponding capacity‐voltage curves of Zn||Cu asymmetric cells in DT0 and DT30.

Zn||Zn symmetric cells and Zn||Cu asymmetric cells are firstly used to assess the electrochemical performance with DT‐containing electrolyte. Galvanostatic charge/discharge (GCD) tests of Zn||Zn symmetric cells containing different concentrations of DT were performed at 1 mA cm^−2^/1 mAh cm^−2^. As shown in **Figure** **3**a, the Zn||Zn symmetric cell containing DT30 shows a significantly longer cycle life of 2200 h. Cells containing DT10 and DT20 also provide long cycle time of 277 and 614 h, respectively. On the contrary, Zn||Zn symmetric cell without DT as the co‐solvent shows sudden short‐circuit after only 135 h. Figure S11 (Supporting Information) provides the corresponding amplified time‐voltage curves of Zn||Zn symmetric cells, in which there are obvious voltage fluctuations in DT0, DT10, and DT20, and the over‐potential in DT30 remains stable after 2200 cycles. Furthermore, at 5 mA cm^−2^/5 mAh cm^−2^, the Zn||Zn symmetric cell containing DT30 can still stably cycle for more than 800 h without any significant voltage fluctuations, which is also much superior compared to the cells with other electrolytes (Figure 3b). Even under 10 mA cm^−2^/10 mAh cm^−2^, the Zn||Zn symmetric cell containing DT30 can show improved stability and reversibility for more than 350 h (Figure 3c). This demonstrates that DT30 inhibits the H_2_O‐induced side reactions and promotes the reversibility of the Zn anode, prolonging the life of the cell. Furthermore, as shown in Figure S12 (Supporting Information), the addition of DT has light effect on the viscosity and ionic conductivity of the electrolyte. It is worth noting that a homogeneous solution cannot be obtained in DT40 (Figure S13, Supporting Information). Therefore, DT30 is selected as the optimized concentration. Figure 3d displays the Zn||Zn symmetric cell containing DT30 also shows superior stability and reversibility under fast structure and morphology change at high current density of 15 mA cm^−2^. While the cell in DT0 suffers from sudden short‐circuit when the current density is increased to 10 mA cm^−2^. Deep charge–discharge testing of Zn||Zn symmetric cells was also carried out to evaluate the stability of Zn anode in DT30 under large structure and morphology changes. As shown in Figure S14a,b (Supporting Information), Zn||Zn symmetric cells containing DT30 can cycle for over 450 h at high depth of discharge (DOD) of 34.2% and over 80 h at 42.7% DOD. The Zn||Cu asymmetric cells were performed to further evaluate the Zn plating/stripping reversibility (Figure 3e). Compared to the DT0‐containing Zn||Cu asymmetric cell, the cell with DT30 shows obviously superb cycling stability, with coulombic efficiency (CE) close to 100% after 600 cycles. Moreover, the polarization voltage of the Zn||Cu cell containing DT30 is stable at 73 mV during cycling (Figure 3f), owing to the improved uniformity of Zn deposition and the suppressed side reactions. However, due to the crazy dendrites growth, the Zn||Cu cell containing DT0 suffers from short‐circuit after 195 cycles.

**Figure 4:**
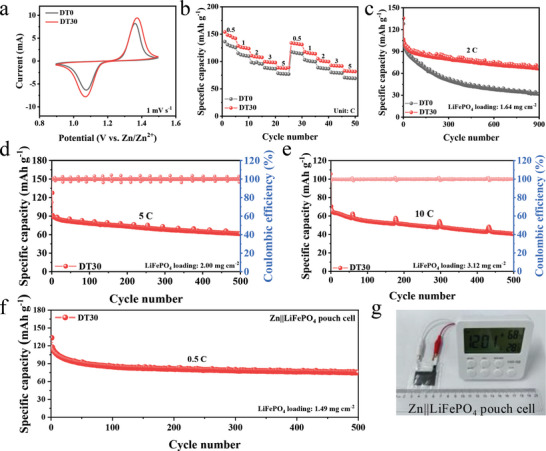
Electrochemical performance evaluation of Zn||LiFePO_4_ full cells at room temperature: a) CV curves, b) rate property, and c) cycling performance in DT0 and DT30. d,e) Cycling performance in DT30 at high rate of 5 C and 10 C. f) Cycling performance of Zn||LiFePO_4_ pouch cell in DT30. g) Digital photograph of Zn||LiFePO_4_ pouch cell powering an electronic meter.

The practical application potential of DT as a co‐solvent is studied using Zn||LiFePO_4_ full cells. As the LiFePO_4_ cathode shows superior reversibility and stability, the commercial LiFePO_4_ was used to highlight the effect of Zn anode on the electrochemical performance of the full cell. Figure S15 (Supporting Information) shows the SEM image of the LiFePO_4_ as nanoparticles. Due to the gelation reaction of the guar gum binder and Zn^2+^ ions, the LiFePO_4_ electrodes prepared using guar gum binder will not undergo dissolution in DT30 and remain structurally stable after 100 cycles (Figure S16, Supporting Information). At the same time, the DT co‐solvent is electrochemically stable in the operating voltage window of AZIBs (Figure S17, Supporting Information). The CV curves in **Figure** **4**a show that the Zn||LiFePO_4_ full cell with DT30 exhibits a pair of redox peaks, corresponding to the Li^+^ ion intercalation/deintercalation into/from the LiFePO_4_ cathode,^[^ ^41^, ^59^ ^]^ which is essentially consistent with the one with DT0. Figure 4b shows the rate performance of Zn||LiFePO_4_ full cells in different electrolytes. The Zn||LiFePO_4_ full cell in DT30 exhibits 146.9, 125.3, 108.5, 98.6, and 87.9 mAh g^−1^ specific capacities from 0.5 to 5 C, which are obviously higher than that of the one using DT0 at different rates. Next, the cycling performance of Zn||LiFePO_4_ full cells in DT0 and DT30 was compared. As shown in Figure 4c, Zn||LiFePO_4_ full cell in DT30 has a high capacity retention of 63% (based on the second cycle) after 900 cycles and a CE of nearly 100%. In contrast, the capacity retention of the cell with DT0 is only 31%, because of the loss of active surface on Zn anode due to drastic side reactions. In addition, the corresponding charge/discharge curves also show the higher capacity and stability of the Zn||LiFePO_4_ full cell in DT30 (Figure S18a,b, Supporting Information). Figure 4d,e displays that at high rates of 5 C and 10 C, Zn||LiFePO_4_ full cells containing DT30 can also cycle stably for more than 500 cycles with the capacity retention of 67% and 61%. The periodic fluctuation in the cycle performance for 5 C and 10 C are due to cyclical fluctuations of the incubator temperature. Zn||LiFePO_4_ pouch cells with the dimension of 2 cm × 3 cm were also prepared using DT30. As shown in Figure 4f, the Zn||LiFePO_4_ pouch cell in DT30 is stably cycled at 0.5 C for more than 500 cycles with 61% capacity retention. And it can successfully drive an electronic meter (Figure 4g). The results demonstrate that DT has superior practicality as a co‐solvent in AZIBs.

**Figure 5:**
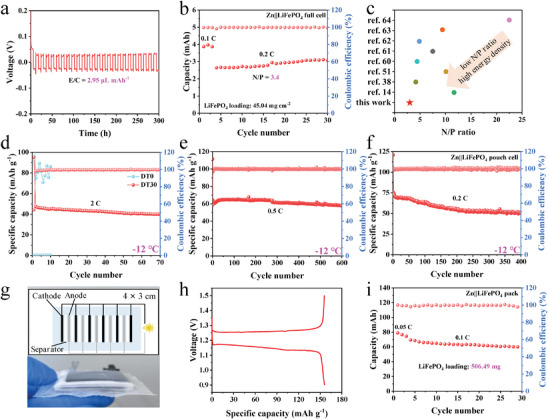
Electrochemical performance evaluation under practical operating conditions: a) Cycling performance of Zn||Zn symmetric cell using DT30 with the E/C ratio of 2.95 µL mAh^−1^ at 1 mA cm^−2^/6 mAh cm^−2^. b) Cycling performance of Zn||LiFePO_4_ full cell using DT30 with the N/P ratio of 3.4 (LiFePO_4_ loading is 45.04 mg cm^−2^). c) Comparison of N/P ratio in the DT30 system with other previously reported excellent electrolyte systems. d) Cycling performance of Zn||LiFePO_4_ full cells in DT0 and DT30 at −12 °C. e) Long‐term cycle performance of Zn||LiFePO_4_ full cell in DT30 at −12 °C. f) Cycling performance of Zn||LiFePO_4_ pouch cell in DT30 at −12 °C. g) Digital photograph of the Zn||LiFePO_4_ pack (inset shows the construction of the pack). h) Typical charge/discharge curve for the first cycle of the Zn||LiFePO_4_ pack. i) Cycling performance of Zn||LiFePO_4_ pack using DT30 with the LiFePO_4_ loading of 506.49 mg.

To achieve practical high energy density of AZIBs, lean electrolyte and low N/P ratio are crucial. Rich in polar −OH groups, DT has excellent hydrophilicity and can powerfully bind H_2_O molecules to prevent H_2_O loss and exhaustion. Therefore, under an extremely low E/C ratio of 2.95 µL mAh^−1^, the Zn||Zn symmetric cell containing DT30 could run normally and stably for more than 300 h (**Figure** **5**a), and Cu||Zn asymmetric cell also operates stably for more than 400 h (Figure S19, Supporting Information). In order to increase the energy density, thinner Zn foils were

used. The Zn||LiFePO_4_ full cell was prepared under an extremely low capacity ratio of negative to positive electrode (N/P) of 3.4 with a thin Zn anode of 20 µm. As shown in Figure 5b, the Zn||LiFePO_4_ full cell exhibits a high initial capacity of 3.88 mAh and maintains at 3.08 mAh after 30 cycles with a CE of 99.3%. This causes by the reason that DT reconstructs the coordination structure of Zn^2+^ ions to improve the reversibility of Zn plating/stripping and suppresses the H_2_O‐induced corrosion reactions, and thus improves the utilization of the Zn anode. Figure 5c compares the N/P ratio in the DT‐based electrolyte system with other previously reported excellent electrolyte systems,^[^ ^14^, ^38^, ^51^, ^60^, ^61^, ^62^, ^63^, ^64^ ^]^ indicating the significantly low N/P ratio has been achieved by facile application of the eco‐friendly DT co‐solvent.

Under low temperature, the frozen electrolyte in the cell internal system causes a remarkable decrease in ionic conductivity and transport kinetics. As discussed above, the DT disrupts the H‐bonds of H_2_O and hinders the orderly arrangement of H_2_O molecules connected by H‐bonds, lowering the freezing point of the electrolyte. Therefore, when the temperature decreases to as low as −12 °C, DT30 electrolyte still provides an acceptable ionic conductivity of 4.44 mS cm^−1^ (Figure S20, Supporting Information). As a result, the Zn||Zn symmetrical cell containing DT30 can cycle normally for more than 200 h at −12 °C (Figure S21, Supporting Information). Cycling performance of Zn||LiFePO_4_ full cells in DT0 and DT30 was also tested at −12 °C. Apparently, the cycling performance is significantly improved with the addition of DT in low temperature (Figure 5d). The DT30‐containing Zn||LiFePO_4_ full cell could cycle stably at 2 C, while the cell in DT0 fails to normal operation due to the freezing of the electrolyte. Moreover, the Zn||LiFePO_4_ full cell containing DT30 achieves an ultra‐long cycle life of over 600 cycles at −12 °C with a capacity retention of 90% (based on the second cycle) and a CE close to 100% (Figure 5e). Figure S22 (Supporting Information) shows the rate performance of the Zn||LiFePO_4_ full cell containing DT30 at −12 °C. The cell achieves a specific capacity of 55 mAh g^−1^ even at 3 C, owing to the improved freezing resistance of DT30. The Zn||LiFePO_4_ pouch cell was also prepared to evaluate the low‐temperature performance of DT30. It shows that the Zn||LiFePO_4_ pouch cell can cycle stably and durably for over 400 cycles with a capacity retention of 67% at −12 °C (Figure 5f). Therefore, DT as an eco‐friendly multifunctional co‐solvent provides a sustainable and effective strategy for the practical application of AZIBs under lean electrolyte, low N/P ratio, and low‐temperature environment.

As a proof‐of‐concept, a parallel‐connected 4 cm × 3 cm Zn||LiFePO_4_ pack consisting of six‐layer LiFePO_4_ cathodes (four of the layers are double‐sided cathodes) and five‐layer Zn anodes was constructed (Figure 5g). The LiFePO_4_ loading of this pack is as high as 506.49 mg. Figure 5h shows the charge/discharge curve for the first cycle of this pack. It shows the similar redox behavior compared to the single Zn||LiFePO_4_ full cell, with an initial specific capacity of 156.0 mAh g^−1^. The Zn||LiFePO_4_ pack provides an initial capacity of up to 79.01 mAh (Figure 5i) and can normally cycle. Therefore, the strong practical potential of DT as a co‐solvent is strongly demonstrated.

## 3 Conclusion

In summary, we propose a natural biomass disaccharide, d‐trehalose (DT), as a multifunctional co‐solvent for AZIBs electrolyte. DT, with abundant polar −OH groups, can massively reconstruct the coordination structure of Zn^2+^ ions and the H‐bonding network of the electrolyte, which has been demonstrated both theoretically and experimentally. On one hand, the coordination interaction between DT and Zn^2+^ ions properly increases the nucleation over‐potential and decreases the nucleation size for flattened Zn deposition. On the other hand, the strong H‐bonds interactions between DT and H_2_O molecules can not only firmly bond H_2_O molecules to decrease the H_2_O activity, but also inhibit the ordered arrangement of the H_2_O molecules under freezing point. As a result, under an extremely low E/C ratio of 2.95 µL mAh^−1^, the Zn||Zn symmetric cell can run normally and stably for more than 300 h; under a significantly low N/P ratio of 3.4, the Zn||LiFePO_4_ full cell exhibits a high capacity of 3.08 mAh after 30 cycles; when the temperature is as low as −12 °C, DT30 electrolyte still provides an acceptable ionic conductivity of 4.44 mS cm^−1^ and the Zn||LiFePO_4_ full cell achieves an ultra‐long cycle life of over 600 cycles. As a proof‐of‐concept, a Zn||LiFePO_4_ pack with LiFePO_4_ loading of 506.49 mg can be achieved. Therefore, a practical and sustainable AZIBs has been achieved by the facile application of the DT co‐solvent.

## 4 Experimental Section

### Preparation of the Electrolyte

d‐trehalose (DT, 99.3%) was purchased from Huiyang Biotechnology Company (China). Zinc sulfate heptahydrate (ZnSO_4_·7H_2_O, 99.0%) was purchased from Sinopharm. Lithium sulfate monohydrate (Li_2_SO_4_·H_2_O, 99.0%) was purchased from Aladdin. The DT‐containing electrolytes were prepared by adding 1.0, 2.0, and 3.0 g of DT to 10 mL 2 M ZnSO_4_ aqueous solution and used to evaluate the symmetric and asymmetric cells. The electrolyte containing 3.0 g DT in 10 mL 1 m ZnSO_4_ + 1 M Li_2_SO_4_ aqueous solution was prepared to evaluate the Zn||LiFePO_4_ full cells. The corresponding electrolytes were noted as DT10, DT20, and DT30, respectively.

### Characterization

FTIR spectra of the electrolytes were performed with a Thermo Scientific Nicolet iS20. The morphologies of Zn electrodes were explored using an emission scanning electron microscope (HITACHI, Regulus8100). ^1^H nuclear magnetic resonance (NMR) spectra were collected on Bruker Avance NEO 400 MHz. Differential scanning calorimeter (DSC) curves were performed on TA DSC25 in the procedure of +20–−40 °C with a cooling and heating rate of 5 °C min^−1^ in the N_2_ atmosphere.

### Electrochemical Measurements

The symmetric and asymmetric cells were assembled with Zn foil, glass fiber separator, and Zn/Cu/Ti foil. Galvanostatic charge/discharge tests were performed with a LAND‐CT3002A battery tester using CR2016 type coin cells at 25 °C. Chronoamperometry (CA) curves were measured at a constant over‐potential of −150 mV for 600 s. The Tafel plots were performed in a three‐electrode system with Zn foil as the working electrode, Pt foil as the counter electrode, and Ag/AgCl as the reference electrode at a scan rate of 1.0 mV s^−1^. Cyclic voltammetry (CV) tests of the Zn||Ti asymmetric cells were performed at a scan rate of 1.0 mV s^−1^ from −0.2 to 0.6 V. Linear sweep voltammetry (LSV) curves were performed at a scan rate of 2.0 mV s^−1^ using a three‐electrode system, with stainless steel (SS) foils served both as the working and counter electrodes and Ag/AgCl as the reference electrode. The ionic conductivity of the electrolytes was measured by electrochemical impedance spectroscopy (EIS) in a frequency range of 100 kHz–0.01 Hz within SS||SS occluded cells. The ionic conductivity was calculated using Equation (1):

(1) $$\sigma \;=\frac{L}{R\;\times \;A}\;$$

where A is the contact area between the separator and the SS electrode (cm^2^), L is the thickness of the separator (cm), and R is the bulk resistance (mΩ). CV, CA, LSV, and EIS electrochemical tests were performed using an electrochemical workstation (Corrtest CS2350M).

### Zn||LiFePO_4_ Full Cell

LiFePO_4_ (P198‐S13) was purchased and used as the cathode material. The LiFePO_4_ working electrode was prepared with LiFePO_4_, conductive carbon black, and guar gum in a weight ratio of 8:1:1 using deionized water as solvent. The mixture was coated onto the carbon cloth (titanium mesh for high LiFePO_4_ loading) and dried at 80 °C for 8 h under vacuum. The Zn||LiFePO_4_ full cells were assembled with LiFePO_4_ cathode, glass fiber separator, and Zn anode. For Zn||LiFePO_4_ pouch cells, the size of the LiFePO_4_ electrode was 2 cm × 3 cm. The assembled full cells were galvanostatically tested over the voltage range of 0.9–1.5 V.

### Molecular Dynamics Simulations

Classic molecular dynamics (MD) simulations were carried out to investigate the electrolyte from the atom/molecule level. One Bulk cases (System1) were built for MD simulations. Case System1 contains 400 ZnSO_4_, 11120 H_2_O, and 176 d‐trehalose molecules. The initial configuration systems were constructed through the software of PACKMOL, and all the molecules were randomly inserted in a cubic simulation box. The OPLSAA force field was employed to describe the ZnSO_4_ and d‐trehalose molecules. The TIP3P force field was employed to describe the H_2_O molecules. For the simulation, an energy minimization was first employed to relax the simulation box. Then, an isothermal‐isobaric (NPT) ensemble with a 1.0 fs time step was employed to optimize the simulation box, where the temperature is set to 298.15 K and the pressure is set to 1.0 atm. The temperature and pressure were kept via the Nose‐Hoover thermostat and Parrinello‐Rahman barostat, respectively. The NPT optimization time was set to 50.0 ns, which is enough long to obtain a stable box size. In all the MD simulations, the motion of atoms/molecules was described by classical Newton's equation, which was solved using the velocity‐verlet algorithm. All the MD simulations were performed by using GROMACS 2021.5 package.^[^ ^65^ ^]^

### Density Functional Theory Calculations

All calculations were carried out with the ORCA 5.0.4 software. The PBE0 functional was used for all calculations, combination with the Grimme's D3(BJ) dispersion correction. The RIJCOSX technique was enabled to significantly speed up the calculation.^[^ ^66^ ^]^ The basis set of def2‐SVP were adopted for the geometry optimization and frequency calculations. The geometries were fully optimized without any structural constraints using the solvation model based on solute electron density (SMD) in water environment. The frequency calculations were carried out at the same level of theory to verify that all structures have no imaginary frequency. The desolvation energy (ΔE) of the optimized molecule was calculated at the PWPB95‐D3(BJ)/def2‐QZVPP calculation level.

## Conflict of Interest

The authors declare no conflict of interest.

## Supporting information

Supporting Information

## Data Availability

Data sharing is not applicable to this article as no new data were created or analyzed in this study.
