# Supplementary material for: Massively Reconstructing Hydrogen Bonding Network and Coordination Structure Enabled by a Natural Multifunctional Co‐Solvent for Practical Aqueous Zn‐Ion Batteries
Source: Adv Sci (Weinh). 2024 Apr 11;11(22):2400336. doi: 10.1002/advs.202400336 (PMC11165558; doi:10.1002/advs.202400336)
Supplement: Supplementary file 1 — Supporting Information [file ADVS-11-2400336-s001.docx]

Supporting Information

Massively reconstructing hydrogen bonding network and coordination structure enabled by a natural multifunctional co-solvent for practical aqueous Zn-ion batteries

Yuanze Yu, Qian Zhang, Pengfei Zhang, Xv Jia, Hongjiang Song, Shengkui Zhong, Jie Liu,*

Y. Yu, P. Zhang, X. Jia, H. Song, J. Liu

Youth Innovation Team of Shandong Higher Education Institutions, College of Chemical Engineering, Qingdao University of Science and Technology, Qingdao 266042, Shandong, P.R. China

E-mail: jie.liu@qust.edu.cn

Q. Zhang

Weifang Key Laboratory of Green Processing of Separator for Chemical Power Sources, School of Chemistry and Engineering, Weifang Vocational College, Weifang 261108, Shandong, China

S. Zhong

College of Marine Science and Technology, Yazhou Bay Innovation Research Institute, Hainan Tropical Ocean University, Sanya 572022, Hainan, P.R. China


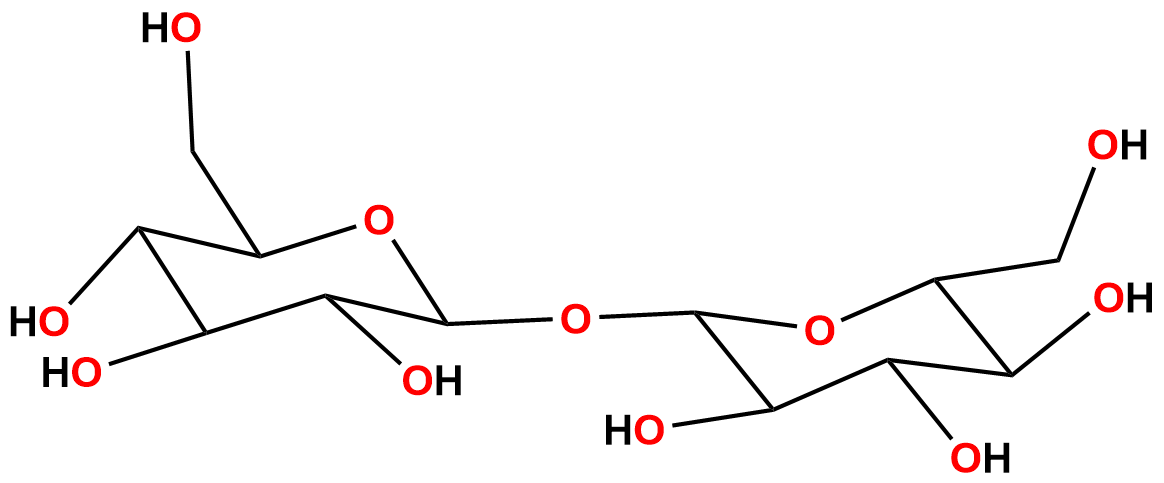


**Figure S1** Molecular structure of DT.


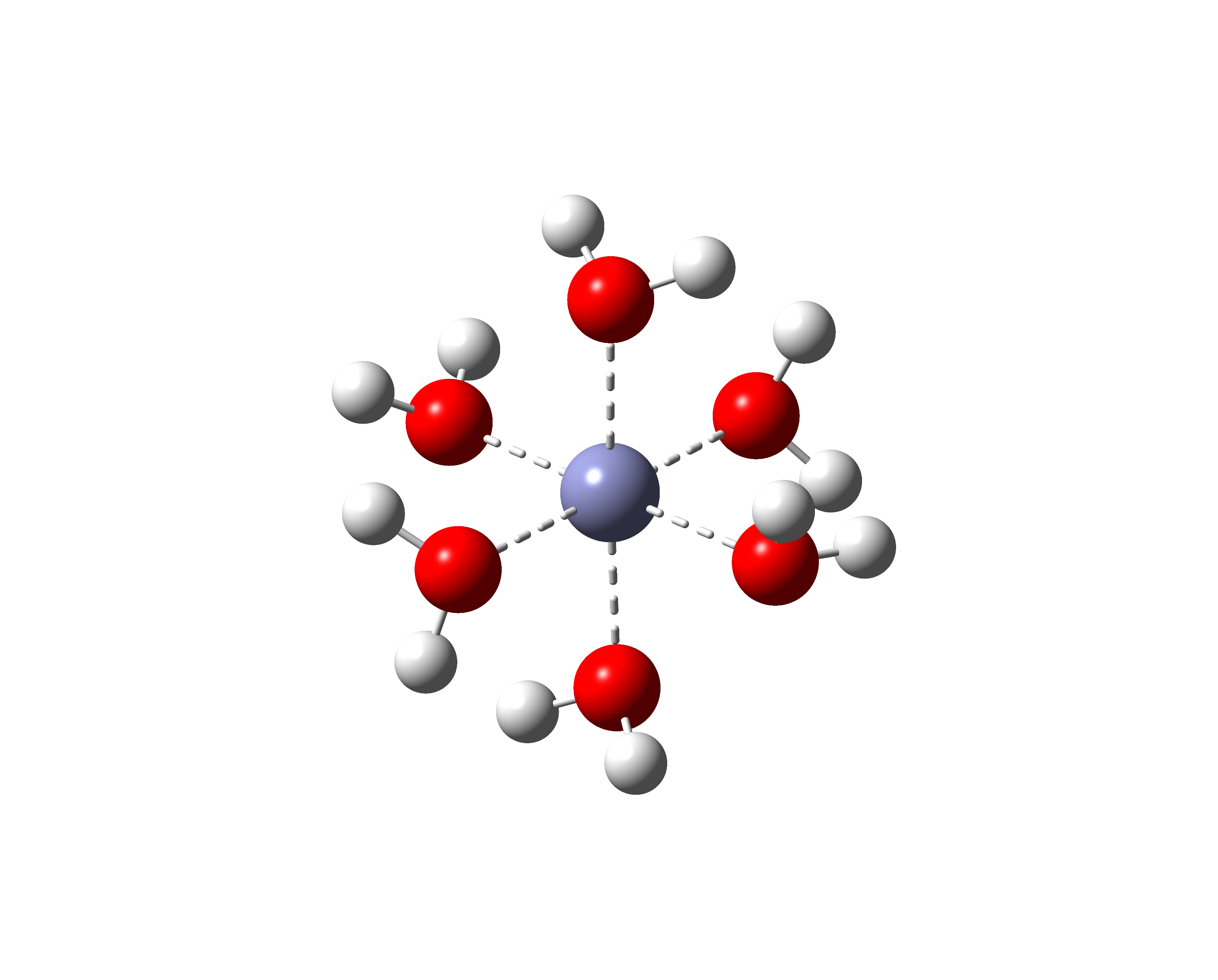


**Figure S2** The Zn^2+^ ion coordination structure in ZnSO_4_ electrolyte.


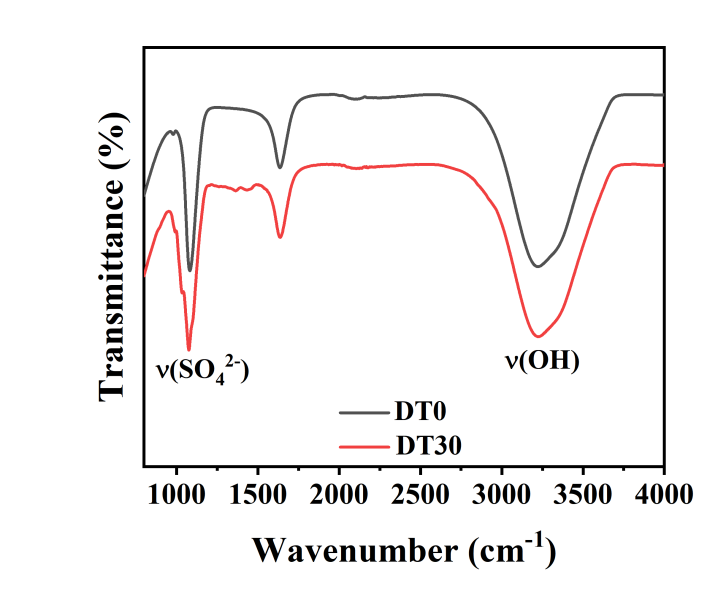


**Figure S3** Full FTIR spectra of different electrolyte.

**Figure S4** ^1^H NMR spectra of different electrolytes.

**Figure S5** DSC curves of DT0 and DT30.

**Figure S6** Digital photo of DT0 and DT30 electrolytes at -12 ℃, demonstrating the freezing resistance of DT30.

**Figure S7** SEM images of Zn electrodes after 50 cycles in (a) DT0 and (b) DT30.


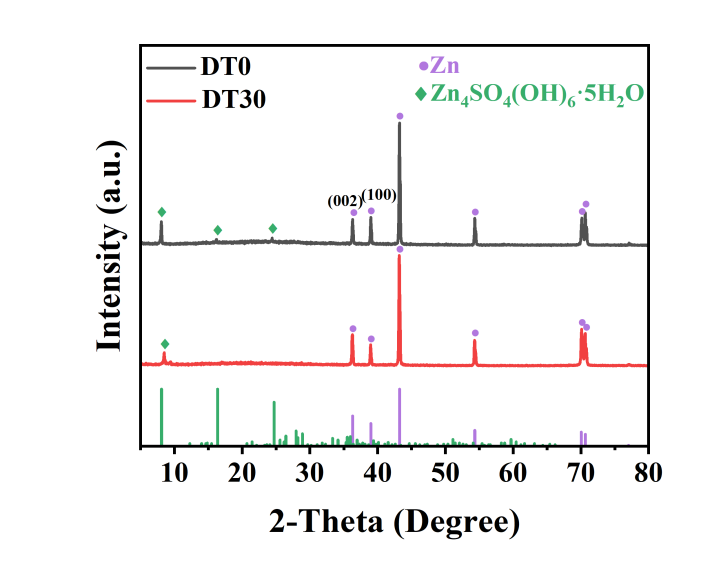


**Figure S8** XRD patterns of the Zn electrodes after 50 cycles in DT0 and DT30.

**Figure S9** Zn nucleation over-potential of Zn||Cu asymmetric cells in DT0 and DT30.

**Figure S10** In-situ optical microscope images of Zn electrodes after plating for 10, 20, 30, 40, 50, and 60 min in (a) DT0 and (b) DT30.

**Figure S11** Typical time-voltage profiles of Zn||Zn symmetric cells in (a) DT0, (b) DT10, (c) DT20, and (d) DT30 at 1 mA cm^-2^/1 mAh cm^-2^.


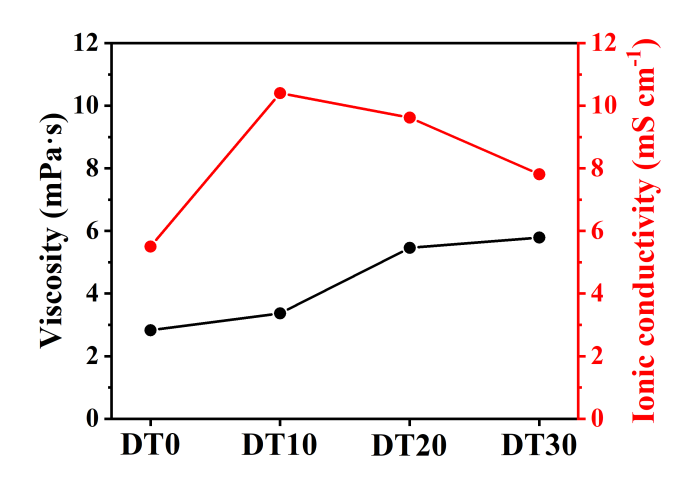


**Figure S12** Viscosity and ionic conductivity of the DT-containing electrolytes.

**Figure S13** Digital photographs of DT-containing ZnSO_4_ electrolytes.

**Figure S14** (a,b) Cycle performance of Zn||Zn symmetric cells in DT30 under high depth of discharge (DOD). The thickness of Zn foil in the DOD test is 20 μm.

**Figure S15** SEM image of the LiFePO_4_.

**Figure S16** (a,b) Digital photos of LiFePO_4_ electrodes before and after immersion in DT30 electrolyte for 48 h. (c,d) SEM images of LiFePO_4_ electrode before and after 100 cycles in DT30.


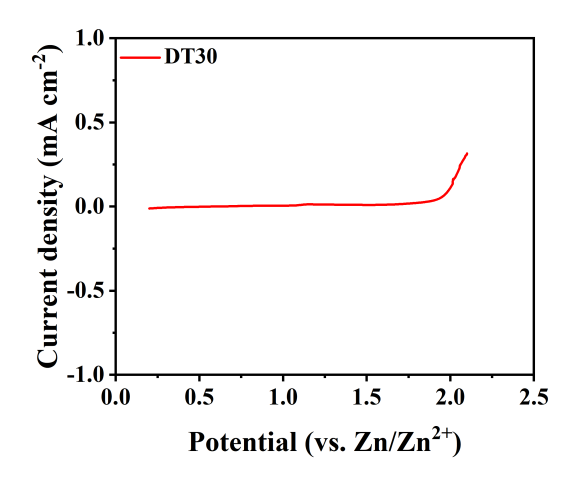


**Figure S17** The electrochemical stability of the DT co-solvent in the voltage window of AZIBs.


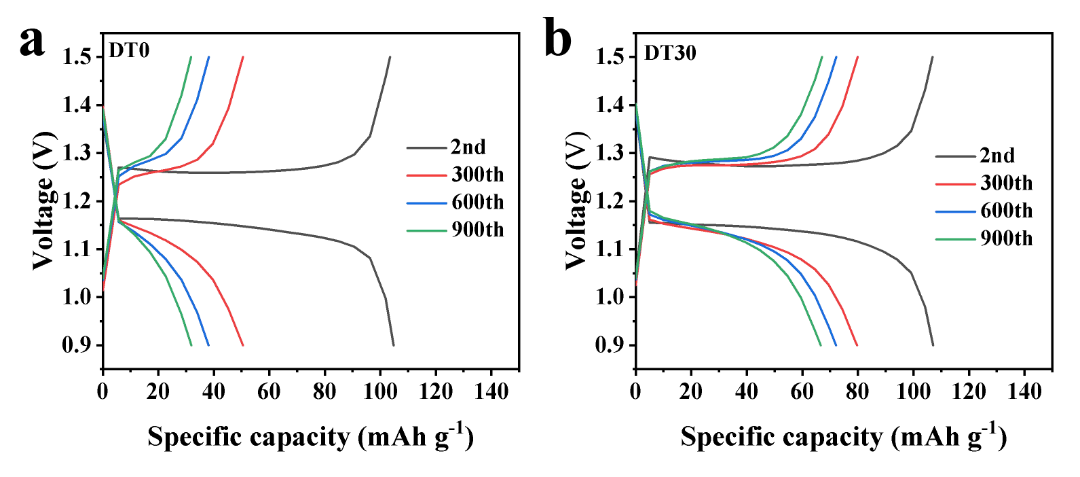


**Figure S18** Typical charge/discharge curves of the Zn||LiFePO_4_ full cells in (a) DT0 and (b) DT30 at 2 C.

**Figure S19** Cycling performance of Cu||Zn asymmetric cell using DT30 with the E/C ratio of 2.95 μL mAh^-1^.


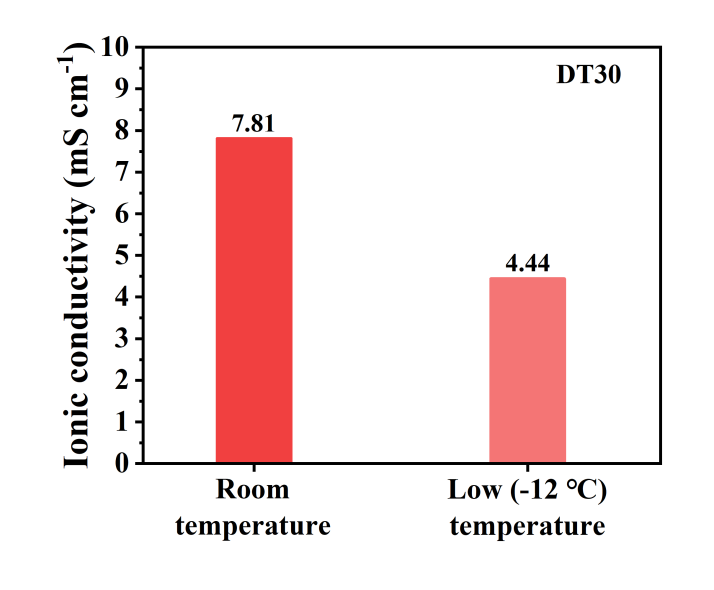


**Figure S20** Ionic conductivity of DT30 at different temperatures.

**Figure S21** Galvanostatic charge/discharge curves of Zn||Zn symmetric cell in DT30 at -12 ℃.

**Figure S22** Rate capability of Zn||LiFePO_4_ full cell in DT30 at -12 ℃.

**Table S1.** Coordination number between Zn^2+^ ions and different ligands.

| Zn^2+^-(O) H_2_O | Zn^2+^-(O) SO_4_^2-^ | Zn^2+^-(O) DT |
| --- | --- | --- |
| 4.20 | 1.67 | 0.17 |

**Table S2.** The number of different H-bonds in DT30

| H_2_O--H_2_O | H_2_O--DT | DT--DT |
| --- | --- | --- |
| 12.5×10^3^ | 3×10^3^ | 0.1×10^3^ |
